# Association Between Muscle Quality and GNRI in Patients with Type 2 Diabetes

**DOI:** 10.3390/nu18020275

**Published:** 2026-01-15

**Authors:** Shinta Yamamoto, Yoshitaka Hashimoto, Fuyuko Takahashi, Moe Murai, Nozomi Yoshioka, Yuto Saijo, Chihiro Munekawa, Hanako Nakajima, Noriyuki Kitagawa, Takafumi Osaka, Ryosuke Sakai, Hiroshi Okada, Naoko Nakanishi, Saori Majima, Emi Ushigome, Masahide Hamaguchi, Michiaki Fukui

**Affiliations:** 1 Department of Endocrinology and Metabolism, Graduate School of Medical Science, Kyoto Prefectural University of Medicine, Kyoto 602-8566, Japan; todayweb@koto.kpu-m.ac.jp (S.Y.);; 2 Department of Diabetes and Endocrinology, Matsushita Memorial Hospital, Moriguchi 570-8540, Japan; 3 Department of Diabetology, Kameoka Municipal Hospital, Kameoka 621-0826, Japan; 4 Department of Endocrinology and Diabetology, Ayabe City Hospital, Ayabe 623-0011, Japan; 5 Department of Diabetes and Metabolism, Osaka Railway Hospital, Osaka 545-0053, Japan; 6 Department of Fundamental Science, Kyoto Institute of Technology, Kyoto 606-0951, Japan

**Keywords:** type 2 diabetes, Geriatric Nutritional Risk Index, muscle quality

## Abstract

**Background:** Type 2 diabetes (T2D) has been linked to impairments in skeletal muscle performance, encompassing reductions in both muscle strength and muscle quality. While malnutrition is a known modifiable factor contributing to muscle quality deterioration, its specific relationship with the Geriatric Nutritional Risk Index (GNRI) in T2D remains underexplored. Using data from 743 participants in the KAMOGAWA-A cohort, this cross-sectional study evaluated the association between muscle quality and GNRI in individuals with type 2 diabetes. **Methods:** Muscle quality was defined as handgrip strength divided by arm lean mass. GNRI was calculated using serum albumin and body mass index. Multiple linear regression models were used to assess associations between GNRI and muscle quality. To account for BMI-related dependency in muscle quality measurements, we derived BMI-adjusted GNRI residuals and performed the same regression analysis to evaluate the stability of the observed relationship beyond BMI-induced confounding. **Results:** In the overall population, GNRI was inversely associated with muscle quality (β = −0.17, *p* < 0.001). Conversely, residual GNRI demonstrated a significant positive association with muscle quality (β = 0.13, *p* < 0.001), especially among men, individuals under 65 years of age, and across all BMI categories. Stratified analyses suggested that the strength and direction of associations varied by age, sex, and glycemic control status. **Conclusions:** The GNRI showed an inverse correlation with muscle quality, whereas residual GNRI showed a consistent positive relationship. These findings suggest that improving nutritional status may support muscle function in T2D, but BMI confounds the interpretation of GNRI in this context.

## 1. Introduction

Chronic dysregulation of glucose metabolism underlies type 2 diabetes (T2D), one of the most prevalent metabolic disorders worldwide. Individuals with T2D are increasingly recognized to be associated with a decline in skeletal muscle mass and muscle strength, such as grip strength, and these associations have been widely reported in the recent literature [1,2,3]. In fact, sarcopenia, which is defined by decline in skeletal muscle mass and muscle strength, is reported to be associated with risk of mortality in individuals with T2D [4]. The mechanisms underlying muscle strength and functional impairment in diabetes are thought to be complex, involving increased fat accumulation in skeletal muscle, accumulation of advanced glycation end products [5], cytokine overproduction, neuropathy, and insulin resistance [6,7,8]. Furthermore, enhanced protein catabolism and mitochondrial dysfunction [9,10] linked to poor glycemic control and chronic hyperglycemia are also considered to play a critical role [11]. 

In addition to muscle mass and strength, indices representing muscle quality also exist, which are assessed by measures such as intramuscular fat content or the ratio of muscle strength to muscle mass. These indicators show strong associations with T2D and its complications [12,13]. Declining muscle quality is linked to all-cause mortality and macrovascular events risk [14,15], suggesting the significance of muscle quality in comprehensive diabetes management. Factors contributing to the decline in muscle quality include insulin resistance [16], aging [17], chronic inflammation [18], physical inactivity [19], smoking [20], and malnutrition [21,22]. Among these factors, malnutrition is a modifiable element, and clarifying the effects of nutritional improvement on muscle quality is clinically highly significant.

However, the association between nutritional status and muscle quality in T2D has not been sufficiently investigated. Therefore, this cross sectional study aimed to clarify the association between nutritional risk, evaluated by the Geriatric Nutritional Risk Index (GNRI), and muscle quality, evaluated by handgrip strength divided by arm lean mass, which can be easily calculated from clinical information. 

## 2. Methods

### 2.1. Study Design and Cohort

This study utilized cohort data from the KAMOGAWA-A study, a prospective observational study [23]. The participants were individuals with T2D who visited the diabetes and endocrinology outpatient clinics at Kyoto Prefectural University of Medicine Hospital, Kameoka City Hospital, and Matsushita Memorial Hospital between January 2015, and May 2024. The study protocol was approved by the Institutional Review Board of Kyoto Prefectural University of Medicine (Approval No.: ERB-C-1876; Date of approval: 27 November 2020). The study adhered to the principles of the Declaration of Helsinki, with informed consent obtained through an opt out approach. The diagnosis of T2D was made by the attending physician at each institution based on existing diagnostic criteria [24]. Individuals who underwent measurements of handgrip strength and body composition were included in the analysis. Individuals with missing blood test data (plasma glucose, serum albumin, and hemoglobin A1c (HbA1c)) were excluded from the analysis.

### 2.2. Data Collection

Sex was recorded as biological sex (male/female) and used as a covariate in all analysis models; information regarding gender was not collected. Diabetes duration was defined as the earliest date among self-reported diagnosis date, initiation of diabetes treatment, or first documented abnormal diabetes-related laboratory test.

Lifestyle factors, including physical activity, smoking, and alcohol consumption, were assessed using standardized self-administered questionnaires. Physical activity was operationalized as exercising one or more times per week; details on exercise modality or intensity were unavailable. Smoking was classified as current or non-smoking, and cumulative smoking exposure was not examined. Alcohol consumption was defined based on habitual drinking behavior. Medication information and a history of cardiovascular disease, including stroke (ischemic or hemorrhagic), heart failure, angina pectoris, coronary artery disease, and myocardial infarction, as well as a history of malignancy, was extracted from electronic medical records. Hypertension was defined as the use of antihypertensive medication or systolic blood pressure ≥140 mmHg/diastolic blood pressure ≥ 90 mmHg. Dyslipidemia was defined as the use of lipid-lowering medication or meeting any of the following criteria: LDL cholesterol ≥ 140 mg/dL, HDL cholesterol < 40 mg/dL, or triglycerides ≥ 150 mg/dL.

Key laboratory parameters (HbA1c, triglycerides, and serum albumin) were measured from blood samples collected after at least 12 h of fasting. Handgrip strength was measured using a Smedley-type hand dynamometer (TTK, Takei Scientific Instruments, Niigata, Japan) for both hands, and the maximum value was used for analysis. Body weight and appendicular skeletal muscle mass were evaluated using a multi-frequency bioelectrical impedance analysis device (InBody 720, 770, or S10, InBody Japan, Tokyo, Japan). Arm lean mass was defined as the average of the right and left measurements.

Body mass index (BMI), muscle quality, GNRI were calculated using the following formulas [25,26]:

$$\begin{array}{c} \mathrm{BMI}\;=\;\mathrm{body}\;\mathrm{weight}\;(\mathrm{kg})/{\mathrm{height}}^{2}\;(m^{2});\;\mathrm{Muscle}\;\mathrm{quality}\;=\;\mathrm{grip}\;\mathrm{strength}\;(\mathrm{kg})/\mathrm{arm}\;\mathrm{lean}\;\mathrm{mass}\;(\mathrm{kg});\;\mathrm{and}\;\mathrm{GNRI}\;=\\ 14.89\;\times \;\mathrm{Serum}\;\mathrm{Albumin}\;(g/\mathrm{dL})\;+\;41.7\;\times \;\mathrm{BMI}/22. \end{array}$$

### 2.3. Statistical Analysis

All statistical analyses were performed using R software (version 4.5.1; R Foundation for Statistical Computing, Vienna, Austria) and RStudio (version 2025.9.2.418; Posit Software, Boston, MA, USA). Statistical significance was assessed using a two-sided threshold of *p* < 0.05. Continuous variables were summarized as means with standard deviations (SD), and categorical variables were summarized as counts with percentages. To evaluate the relationship between the GNRI and muscle quality, scatter plots were generated and stratified by categories of age (<65, 65–74, ≥75 years), sex, HbA1c (<6.5%, 6.5–8%, ≥8%), and BMI (<22, 22–25, >25 kg/m^2^). Simple and multiple linear regression analyses were then conducted to characterize standardized regression coefficients (β) and 95% confidence intervals (CIs). Because muscle quality includes muscle mass in its calculation and GNRI incorporates BMI as a component, a residual GNRI was derived by removing the effect of BMI, and similar analyses were performed using this variable. The following models were constructed: Model 1: Crude; Model 2: Adjusted for age and sex; Model 3: Additionally adjusted for HbA1c, hypertension, dyslipidemia, cancer, history of cardiovascular disease, smoking, alcohol consumption, physical activity, history of oral antidiabetic medication (biguanide, SGLT2 inhibitor, GLP-1 receptor agonist) use and diabetes duration. 

## 3. Result

**Figure 1:**
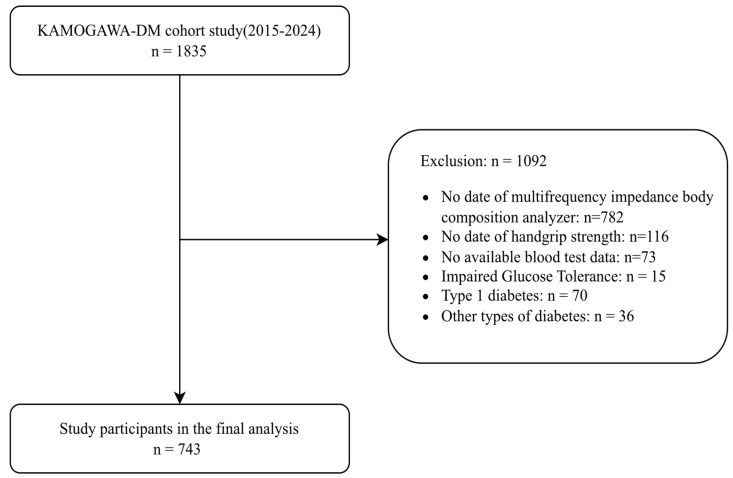
Flow diagram showing the selection of the study population.

From January 2015 to May 2024, 1835 individuals were enrolled in the KAMOGAWA-A cohort. After applying the exclusion criteria, 1092 individuals were excluded, and the final analysis included 743 individuals (Figure 1). 

In this analysis, 743 participants were included; 435 were men and 308 were women. The mean age and diabetes duration were 67.7 years (SD 11.5) and 15.6 years (SD 11.1). The mean BMI, arm lean mass and handgrip strength were 24.5 kg/m^2^ (SD 4.5), 2.3 kg (SD 0.6), and 28.2 kg (SD 9.3), respectively. In addition, the mean muscle quality was 12.5 (SD 2.6) and the mean GNRI was 109.3 (SD 10.2). Hypertension, dyslipidemia, cancer, and cardiovascular disease were present in 57.2%, 56.1%, 13.1%, and 19.8% of participants, respectively (Table 1).

**Figure 2:**
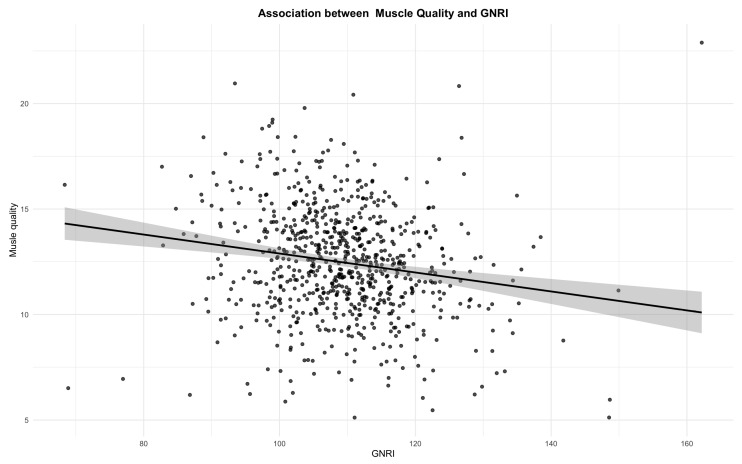
Association between GNRI and muscle quality in patients with type 2 diabetes (R = 0.17, *p* < 0.001).

In the overall analysis, GNRI showed an inverse association with muscle quality (β = –0.17, 95% CIs −0.25 to −0.10, *p* < 0.001) (Figure 2).

A scatter plot showing the relationship between GNRI and muscle quality. The blue line represents the linear regression fit, and the gray area indicates the 95% confidence interval.

This inverse association was consistently observed across age categories: <65 years (β = −0.20, 95% CIs −0.32 to −0.08, *p* < 0.001), 65–74 years (β = −0.20, 95% CIs −0.32 to −0.08, *p* < 0.001), and ≥75 years (β = −0.17, 95% CIs −0.30 to −0.03, *p* = 0.01) (Table 2 and Supplementary Figure S1). When stratified by sex, the association remained significant in females (β = −0.32, 95% CIs −0.42 to −0.21, *p* < 0.001) but not in males (β = −0.06, 95% CIs −0.15 to 0.03, *p* = 0.21) (Table 2 and Supplementary Figure S3). Across HbA1c categories, no significant associations were identified for <6.5% or 6.5–7.9%, whereas a clear inverse association was observed among participants with HbA1c ≥ 8.0% (β = −0.33, 95% CIs −0.46 to −0.20, *p* < 0.001) (Table 2 and Supplementary Figure S2). Across BMI categories, no significant associations were identified (Table 2 and Supplementary Figure S4).

**Figure 3:**
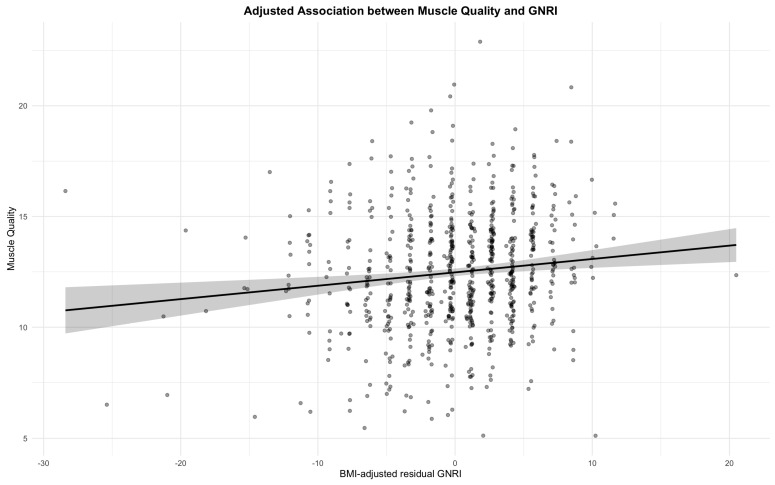
Association between BMI-adjusted residual GNRI and muscle quality in patients with type 2 diabetes.

Analyses using BMI-adjusted residual GNRI demonstrated a positive association with muscle quality across all models, with the fully adjusted model showing β = 0.12 (95% CIs 0.05 to 0.19, *p* = 0.001) (Figure 3). 

A scatter plot showing the relationship between BMI-adjusted residual GNRI and muscle quality. The blue line represents the linear regression fit, and the gray area indicates the 95% confidence interval. 

In age-stratified analyses, participants younger than 65 years showed a significant positive association (β = 0.17, 95% CIs 0.04 to 0.29, *p* = 0.01), whereas the associations were not significant in those aged 65–74 years (β = 0.08, 95% CIs −0.03 to 0.20, *p* = 0.15) or those aged ≥75 years (β = 0.11, 95% CIs −0.03 to 0.25, *p* = 0.13) (Table 3 and Supplementary Figure S5). Among male, residual GNRI remained positively associated with muscle quality after full adjustment (β = 0.12, 95% CIs 0.02 to 0.22, *p* = 0.02), whereas no significant association was found in women (β = 0.09, 95% CIs −0.02 to 0.20, *p* = 0.12) (Table 3 and Supplementary Figure S6). Significant positive associations were also observed across BMI strata: <22 kg/m^2^ (β = 0.21, 95% CIs 0.06 to 0.35, *p* = 0.005), 22–25 kg/m^2^ (β = 0.14, 95% CIs 0.01 to 0.27, *p* = 0.04), and >25 kg/m^2^ (β = 0.13, 95% CIs 0.01 to 0.25, *p* = 0.04) (Table 3 and Supplementary Figure S7). In HbA1c-stratified analyses, significant associations were observed for HbA1c < 6.5% (β = 0.34, 95% CIs 0.17 to 0.50, *p* < 0.001) and 6.5–7.9% (β = 0.11, 95% CIs 0.01 to 0.21, *p* = 0.04), whereas no association was identified for HbA1c ≥ 8.0% (β = 0.09, 95% CIs −0.05 to 0.23, *p* = 0.19) (Table 3 and Supplementary Figure S8).

## 4. Discussion

This study assessed the relationship between the GNRI and muscle quality in individuals with type 2 diabetes. In the overall analysis, GNRI showed a significant inverse association with muscle quality, with a more pronounced relationship in female and in participants with HbA1c levels ≥ 8.0%. In contrast, analyses using BMI-adjusted residual GNRI demonstrated consistent positive associations across all models, particularly among participants younger than 65 years, male, and across all BMI categories. Although the observed standardized β coefficients indicate modest effect sizes, this magnitude is consistent with population-based studies examining nutritional status and functional outcomes and should be interpreted as a meaningful association at the population level rather than a large effect at the individual level. Previous studies have reported that GNRI correlates positively with muscle strength indicators, such as handgrip strength, and with muscle mass indices, including skeletal muscle mass index, in various populations including individuals with T2D. These findings have been documented in several cohort studies [27,28,29,30]. Notably, Takahashi et al. [27] reported that GNRI shows a stronger correlation with muscle mass than with muscle strength. This supports our finding that GNRI exhibited a negative association with muscle quality. Furthermore, the positive association observed with residual GNRI highlights the importance of accounting for the influence of BMI. As GNRI incorporates body weight as a component, it is strongly affected by overall body composition, whereas residual GNRI reflects nutritional status independent of BMI. These results suggest that improvements in GNRI may not directly translate to improvements in muscle quality; however, improvement in nutritional status may still contribute to better muscle quality, particularly when the influence of body size is accounted for. In subgroup analyses, the adjusted models showed significant associations only in participants younger than 65 years and in males, whereas no significant association was observed among individuals with HbA1c ≥ 8.0%. In the HbA1c-stratified analysis, BMI-adjusted residual GNRI was not significantly associated with muscle quality in participants with poor glycemic control. This finding suggests that in individuals with HbA1c ≥ 8.0%, the adverse effects of chronic hyperglycemia on skeletal muscle may be more prominent than the influence of nutritional status. Persistent hyperglycemia has been shown to induce oxidative stress, mitochondrial dysfunction, chronic inflammation, and the accumulation of advanced glycation end products, all of which can impair muscle mass and muscle function. Under such metabolic conditions, variability in nutritional status captured by residual GNRI may have a limited or masked association with muscle quality. Alternatively, the lack of a significant association in this subgroup may be partly explained by reduced statistical power or greater heterogeneity in clinical characteristics, including differences in diabetes duration, comorbidities, or treatment regimens. Therefore, these results should not be interpreted as evidence that nutritional status is unimportant in individuals with poor glycemic control, but rather that the relationship between nutritional status and muscle quality may be modified by the severity of hyperglycemia. Given the cross-sectional nature of the present study, these subgroup findings should be regarded as exploratory, and future longitudinal studies are required to clarify the relative contributions of hyperglycemia and nutritional status to muscle quality in individuals with poorly controlled T2D. Among older adults, age-related declines in muscle mass and muscle strength may attenuate or obscure the contribution of nutritional status. In participants with poor glycemic control, chronic hyperglycemia may lead to oxidative stress and the accumulation of advanced glycation end products, which contribute to reductions in muscle mass and muscle strength [31]. In addition, sex-specific biological factors such as hormonal regulation, including the effects of estrogen, may influence muscle health in female [32]. These findings suggest that factors beyond nutritional status may contribute to muscle quality in certain subpopulations, and that the relationship between nutritional status and muscle quality may be modified by age, sex, and metabolic conditions, indicating the need for a multifaceted approach to muscle preservation.

This study has several limitations. First, the cross-sectional design does not allow causal inference, and whether improvements in GNRI lead to better muscle quality remains unclear. Second, although residual GNRI showed a positive association with muscle quality, GNRI values in clinical practice may reflect changes in body composition, and careful interpretation is required. In addition, because more than half of the initially enrolled participants were excluded from the analysis due to missing data, selection bias may have occurred. The included individuals may have different clinical characteristics compared to the general population with type 2 diabetes, especially as they were recruited from tertiary care centers. This may limit the generalizability of our findings. Third, the assessment of appendicular lean mass was based on bioelectrical impedance analysis, which provides an indirect estimate and may be influenced by hydration status, fat distribution, and metabolic conditions. In particular, altered fluid balance in individuals with diabetes may have affected impedance measurements and introduced some degree of inaccuracy. Fourth, muscle quality can be defined not only by functional measures but also by imaging-based approaches that assess intramuscular fat infiltration (myosteatosis) using CT or MRI [33]. Although imaging-based assessments may more directly capture qualitative structural changes in muscle, they could not be implemented in the present study due to constraints related to cost and availability. Therefore, muscle quality was evaluated using a functional indicator based on handgrip strength normalized to arm lean mass, which may have influenced the interpretation of the results. Physical activity was assessed as the presence or absence of regular exercise, and detailed information regarding exercise type, intensity, or duration was not available. As different forms of exercise may differentially affect muscle quality, residual confounding related to physical activity cannot be excluded. Finally, we did not evaluate other nutritional markers, and further studies are needed to compare GNRI with alternative indices of nutritional status.

## 5. Conclusions

In conclusion, this study demonstrated the relationship between GNRI and muscle quality in individuals with T2D. While GNRI showed an inverse association with muscle quality, BMI-adjusted residual GNRI was positively associated with muscle quality. These findings suggest that improving nutritional status may contribute to the maintenance or enhancement of muscle quality, although factors other than undernutrition should also be considered in clinical management.

## Figures and Tables

**Table 1:** Characteristics of study participants.

Number of Participants	743
Sex (male/female), n	435/308
Age, years	67.7 (11.5)
Duration diabetes, years	15.6 (11.1)
Height, m	1.6 (0.1)
Weight, kg	63.8 (13.4)
BMI, kg/m^2^	24.5 (4.5)
ASM, kg	18.3 (4.3)
Arm skeletal muscle mass (right/left), kg	2.3 (0.6)/2.3 (0.6)
Hand grip strength, kg	28.18 (9.31)
Muscle quality	12.5 (2.6)
Albumin, g/dL	4.2 (0.4)
Glucose, mg/dL	153.9 (51.5)
Hemoglobin A1c, %	7.5 (1.3)
GNRI	109.3 (10.2)
Smoking status, n (%)	208 (28.0)
Alcohol consumption, n (%)	385 (51.8)
Exercise habits, n (%)	297 (40.0)
Hypertension, n (%)	425 (57.2)
Dyslipidemia, n (%)	417 (56.1)
Cancer, n (%)	97 (13.1)
Cardiovascular disease, n (%)	147 (19.8)
Biguanide, n (%)	357 (48.0)
SGLT2 inhibitor, n (%)	208 (28.0)
GLP-1 receptor agonist, n (%)	133 (17.9)

Data are presented as means (standard deviations) for continuous variables and numbers (percentages) for categorical variables. Abbreviations: ASM, appendicular skeletal muscle mass; BMI, body mass index; GNRI, Geriatric Nutritional Risk Index; GLP-1, Glucagon-like peptide-1; SGLT2, Sodium-Glucose Cotransporter 2.

**Table 2:** Association between GNRI and muscle quality in overall and subgroup analyses.

		Standardized β (95% CI)	*p* Value	Interaction *p*
All		−0.17 (−0.25–−0.10)	<0.001	
Subgroup	Age			0.88
	<65 years	−0.20 (−0.32–−0.08)	<0.001	
	65–74 years	−0.20 (−0.32–−0.08)	<0.001	
	≥75 years	−0.17 (−0.30–−0.03)	0.01	
	Sex			<0.001
	Male	−0.06 (−0.15–0.03)	0.21	
	Female	−0.32 (−0.42–−0.21)	<0.001	
	BMI			0.39
	<22 kg/m^2^	0.03 (−0.10–0.16)	0.64	
	22–25 kg/m^2^	0.05 (−0.08–0.18)	0.46	
	>25 kg/m^2^	−0.07 (−0.19–0.05)	0.25	
	Hemoglobin A1c			0.052
	<6.5%	−0.14 (−0.30–0.03)	0.1	
	6.5–7.9%	−0.09 (−0.19–0.01)	0.08	
	≥8.0%	−0.33 (−0.46–−0.20)	<0.001	

Abbreviations: BMI, body mass index. Standardized β coefficients were derived from simple linear regression models evaluating the association between the Geriatric Nutritional Risk Index (GNRI) and muscle quality. Subgroup analyses were performed according to age, sex, BMI, and hemoglobin A1c categories. Data are shown as standardized β (95% confidence intervals) with corresponding *p* values.

**Table 3:** Association between BMI-adjusted GNRI (residual GNRI) and muscle quality in overall and subgroup analyses.

		Model 1		Model 2		Model 3		
		Standardized β (95% CI)	*p* Value	Standardized β (95% CI)	*p* Value	Standardized β (95% CI)	*p* Value	Interaction *p*
All		0.16 (0.09–0.23)	<0.001	0.12 (0.05–0.19)	<0.001	0.12 (0.05–0.19)	0.001	
Subgroup	Age							0.98
	<65 years	0.26 (0.14–0.38)	<0.001	0.16 (0.04–0.28)	0.01	0.17 (0.04–0.29)	0.01	
	65–74 years	0.15 (0.03–0.27)	0.02	0.11 (−0.01–0.22)	0.06	0.08 (−0.03–0.20)	0.15	
	≥75 years	0.09 (−0.04–0.23)	0.17	0.10 (−0.03–0.23)	0.14	0.11 (−0.03–0.25)	0.13	
	Sex							0.75
	Male	0.11 (0.02–0.21)	0.02	0.13 (0.03–0.23)	0.01	0.12 (0.02–0.22)	0.02	
	Female	0.19 (0.08–0.30)	<0.001	0.08 (−0.03–0.18)	0.14	0.09 (−0.02–0.20)	0.12	
	BMI							0.43
	<22 kg/m^2^	0.16 (0.03–0.29)	0.02	0.17 (0.04–0.30)	0.01	0.21 (0.06–0.35)	0.005	
	22–25 kg/m^2^	0.13 (−0.00–0.26)	0.052	0.12 (−0.02–0.25)	0.09	0.14 (0.01–0.27)	0.04	
	>25 kg/m^2^	0.24 (0.12–0.35)	<0.001	0.12 (−0.00–0.24)	0.051	0.13 (0.01–0.25)	0.04	
	Hemoglobin A1c							0.02
	<6.5%	0.39 (0.24–0.55)	<0.001	0.34 (0.18–0.49)	<0.001	0.34 (0.17–0.50)	<0.001	
	6.5–7.9%	0.17 (0.07–0.27)	<0.001	0.12 (0.02–0.22)	0.02	0.11 (0.01–0.21)	0.04	
	≥8.0%	0.03 (−0.11–0.17)	0.64	0.04 (−0.09–0.17)	0.54	0.09 (−0.05–0.23)	0.19	

Abbreviations: BMI, body mass index. Standardized β coefficients were derived from linear regression models evaluating the association between BMI-adjusted GNRI (residual GNRI) and muscle quality. Model 1 represents the crude analysis; Model 2 was adjusted for age and sex; and Model 3 was further adjusted for hemoglobin A1c, hypertension, dyslipidemia, cancer, cardiovascular disease, smoking, alcohol consumption, exercise habits, history of oral antidiabetic medication (biguanide, SGLT2 inhibitor, GLP-1 receptor agonist) and diabetes duration. Subgroup analyses were stratified by age, sex, BMI, and hemoglobin A1c categories. Data are presented as standardized β (95% confidence intervals) with corresponding *p* values.

## Data Availability

Data from this study are available from the corresponding author upon reasonable request due to ethical restrictions and the protection of participant privacy.

## Supplementary Materials

The following supporting information can be downloaded at: https://www.mdpi.com/article/10.3390/nu18020275/s1, Supplementary Figure S1. Adjusted association between GNRI and muscle quality stratified by age groups. Scatter plots showing the relationship between residual GNRI and muscle quality across age groups: <65 years, 65–74 years, and ≥75 years. Supplementary Figure S2. Association between GNRI and muscle quality stratified by HbA1c categories. Scatter plots showing the association between GNRI and muscle quality across three HbA1c strata: <6.5%, 6.5–7.9%, and ≥8.0%. Supplementary Figure S3. Association between GNRI and muscle quality stratified by sex. Sex stratified analysis of the association between GNRI and muscle quality. Supplementary Figure S4. Association between GNRI and muscle quality stratified by BMI categories. Scatter plots of the relationship between GNRI and muscle quality across BMI groups: <22 kg/m^2^, 22–25 kg/m^2^, and >25 kg/m^2^. Supplementary Figure S5. Adjusted association between BMI-adjusted residual GNRI and muscle quality stratified by age groups. Scatter plots showing the relationship between BMI-adjusted residual GNRI and muscle quality across age groups: <65 years, 65–74 years, and ≥75 years. Supplementary Figure S6. Association between BMI-adjusted residual GNRI and muscle quality stratified by HbA1c categories. Scatter plots showing the association between BMI-adjusted residual GNRI and muscle quality across three HbA1c strata: <6.5%, 6.5–7.9%, and ≥8.0%. Supplementary Figure S7. Association between BMI-adjusted residual GNRI and muscle quality stratified by sex. Sex stratified analysis of the association between BMI-adjusted residual GNRI and muscle quality. Supplementary Figure S8. Association between BMI-adjusted residual GNRI and muscle quality stratified by BMI categories. Scatter plots of the relationship between BMI-adjusted residual GNRI and muscle quality across BMI groups: <22 kg/m^2^, 22–25 kg/m^2^, and >25 kg/m^2^. Table S1. Association between BMI-adjusted GNRI (residual GNRI) and muscle quality in overall and subgroup analyses with FDR-adjusted *p* values.
