# Supplementary material for: Association Between Muscle Quality and GNRI in Patients with Type 2 Diabetes
Source: Nutrients. 2026 Jan 15;18(2):275. doi: 10.3390/nu18020275 (PMC12844626; doi:10.3390/nu18020275)
Supplement: Supplementary file 1 [file nutrients-18-00275-s001.zip › Supplemental Table 1.pdf]

Supplemental Table S1. Association between BMI-adjusted GNRI (residual GNRI) and muscle quality in overall and subgroup analyses with FDR-adjusted p values

		Model 3		
		Standardized β (95% CI)	p value	FDR-adjusted p value
All		0.13 (0.06 – 0.20)	<0.001	-
Subgroup	Age			
	<65 years	0.15 (0.03 – 0.28)	0.01	0.028
	65-74 years	0.10 (–0.01 – 0.21)	0.09	0.11
	≥75 years	0.12 (–0.01 – 0.26)	0.08	0.11
	Sex			
	Male	0.14 (0.04 – 0.24)	0.01	0.028
	Female	0.08 (–0.03 – 0.19)	0.14	0.15
	BMI			
	<22 kg/m^2	0.22 (0.08 – 0.37)	0.002	0.011
	22–25 kg/m^2	0.14 (0.01 – 0.28)	0.04	0.07
	>25 kg/m^2	0.12 (0.00 – 0.24)	0.049	0.08
	Hemoglobin A1c			
	<6.5%	0.36 (0.20 – 0.53)	<0.001	<0.001
	6.5–7.9%	0.11 (0.01 – 0.21)	0.03	0.07
	≥8.0%	0.08 (–0.06 – 0.21)	0.26	0.26

Abbreviations: BMI, body mass index; GNRI, Geriatric Nutritional Risk Index; FDR, false discovery rate.

Standardized β coefficients were derived from linear regression models evaluating the association between BMI-adjusted GNRI (residual GNRI) and muscle quality. Model 3 was adjusted for age, sex, hemoglobin A1c, hypertension, dyslipidemia, cancer, cardiovascular disease, smoking, alcohol

consumption, exercise habits, and diabetes duration. Subgroup analyses were stratified by age, sex, BMI, and hemoglobin A1c categories. Data are presented as standardized β (95% confidence intervals) with corresponding FDR-adjusted p values. P values for subgroup analyses were adjusted for multiple comparisons using the Benjamini–Hochberg false discovery rate method.
