# Supplementary material for: Association Between Muscle Quality and GNRI in Patients with Type 2 Diabetes
Source: Nutrients. 2026 Jan 15;18(2):275. doi: 10.3390/nu18020275 (PMC12844626; doi:10.3390/nu18020275)
Supplement: Supplementary file 1 [file nutrients-18-00275-s001.zip › Supplementary Figure.pdf]

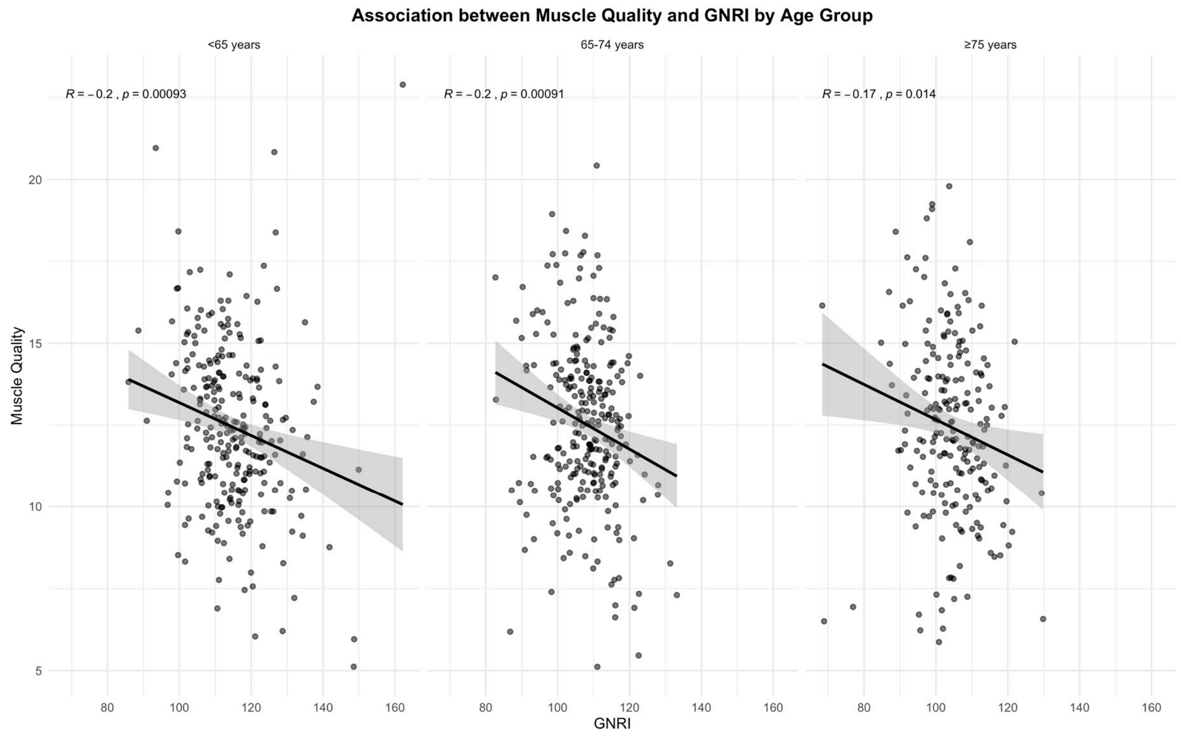

Supplementary Fig. S1 Adjusted association between GNRI and muscle quality stratified by age groups. Scatter plots showing the relationship between residual GNRI and muscle quality across age groups: <65 years, 65–74 years, and ≥75 years.

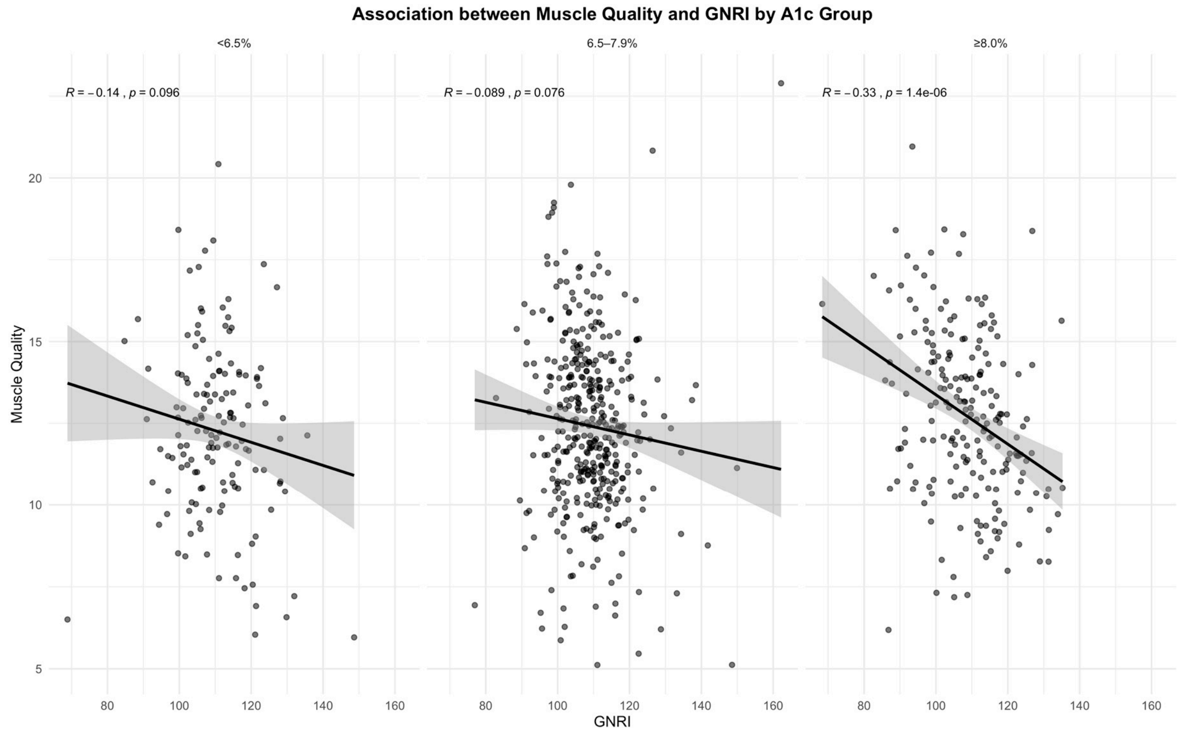

Supplementary Fig. S2 Association between GNRI and muscle quality stratified by HbA1c categories. Scatter plots showing the association between

GNRI and muscle quality across three HbA1c strata: <6.5%, 6.5–7.9%, and ≥8.0%.

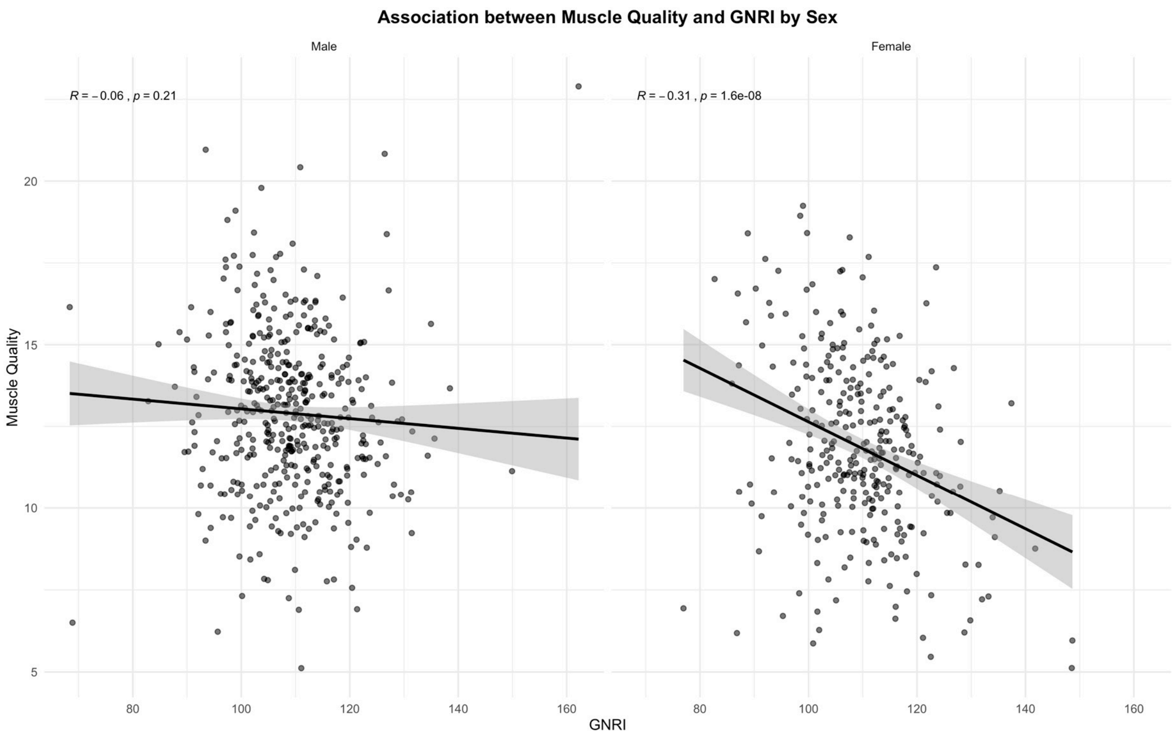

Supplementary Fig. S3 Association between GNRI and muscle quality stratified by sex. Sex stratified analysis of the association between GNRI and muscle quality.

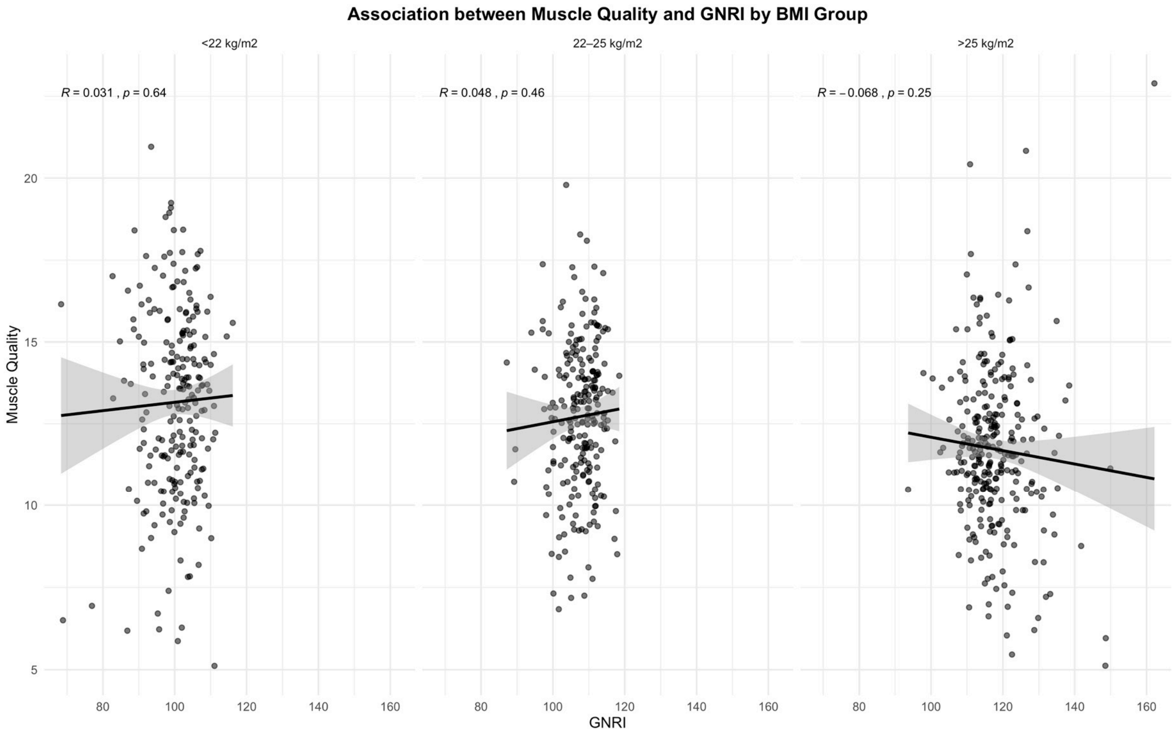

Supplementary Fig. S4 Association between GNRI and muscle quality stratified by BMI categories. Scatter plots of the relationship between GNRI and muscle quality across BMI groups: <22 kg/m², 22–25 kg/m², and >25 kg/m².

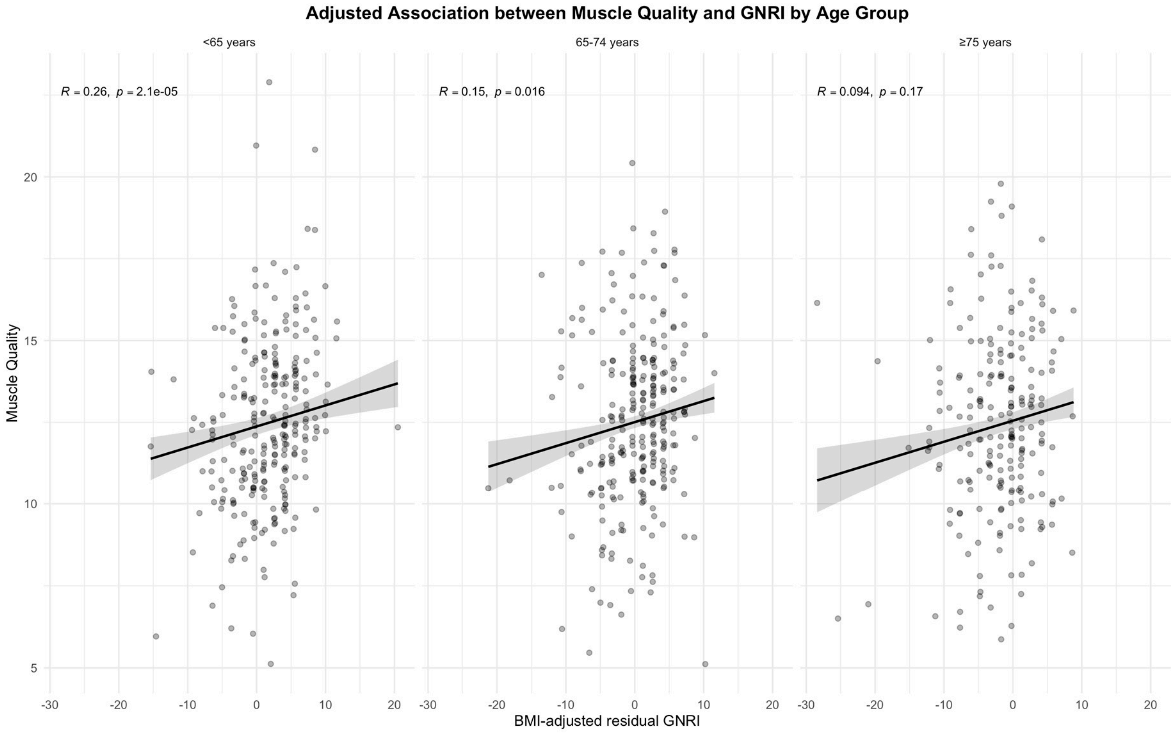

Supplementary Fig. S5 Adjusted association between BMI-adjusted residual GNRI and muscle quality stratified by age groups. Scatter plots showing the relationship between BMI-adjusted residual GNRI and muscle quality across age groups: <65 years, 65–74 years, and ≥75 years.

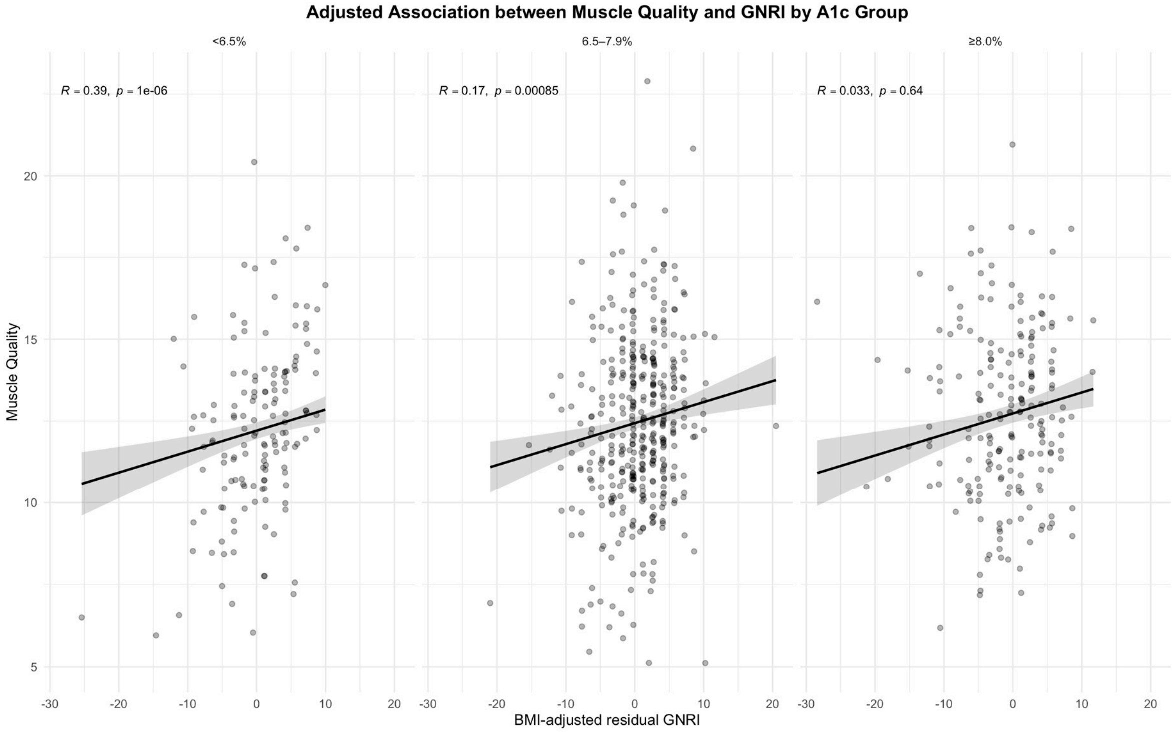

Supplementary Fig. S6 Association between BMI-adjusted residual GNRI and muscle quality stratified by HbA1c categories. Scatter plots showing the association between BMI-adjusted residual GNRI and muscle quality across three HbA1c strata: <6.5%, 6.5–7.9%, and ≥8.0%.

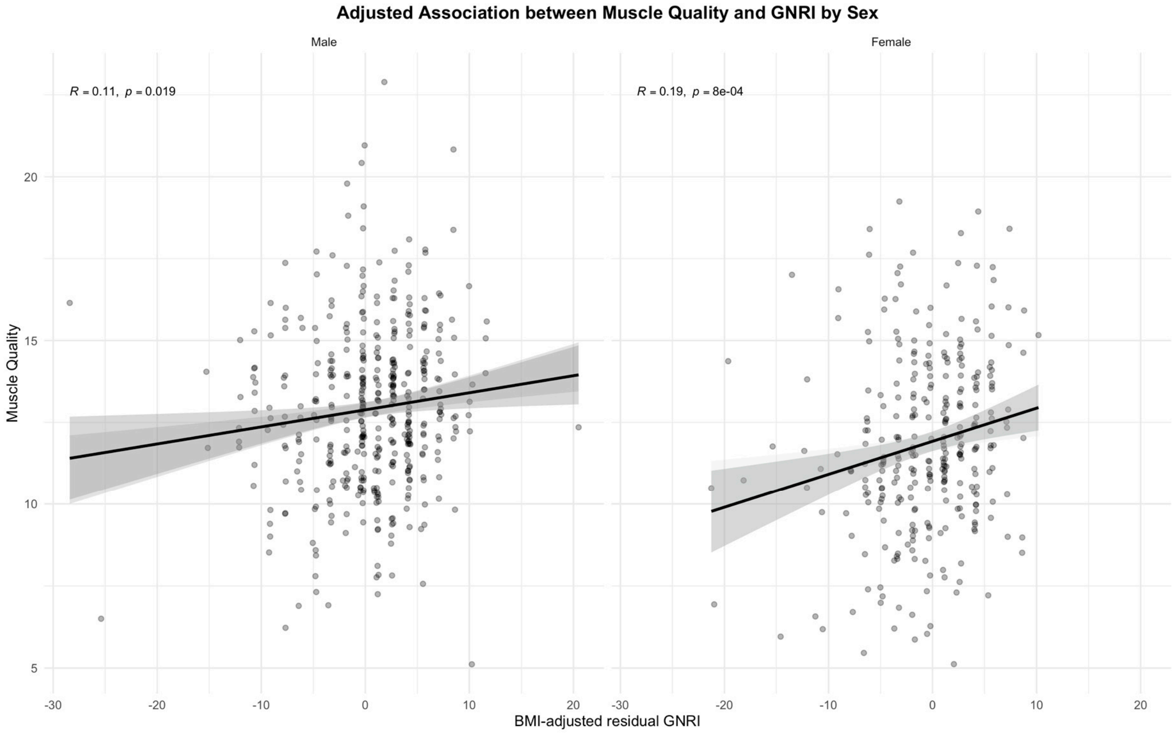

Supplementary Fig. S7 Association between BMI-adjusted residual GNRI and muscle quality stratified by sex. Sex stratified analysis of the association between BMI-adjusted residual GNRI and muscle quality.

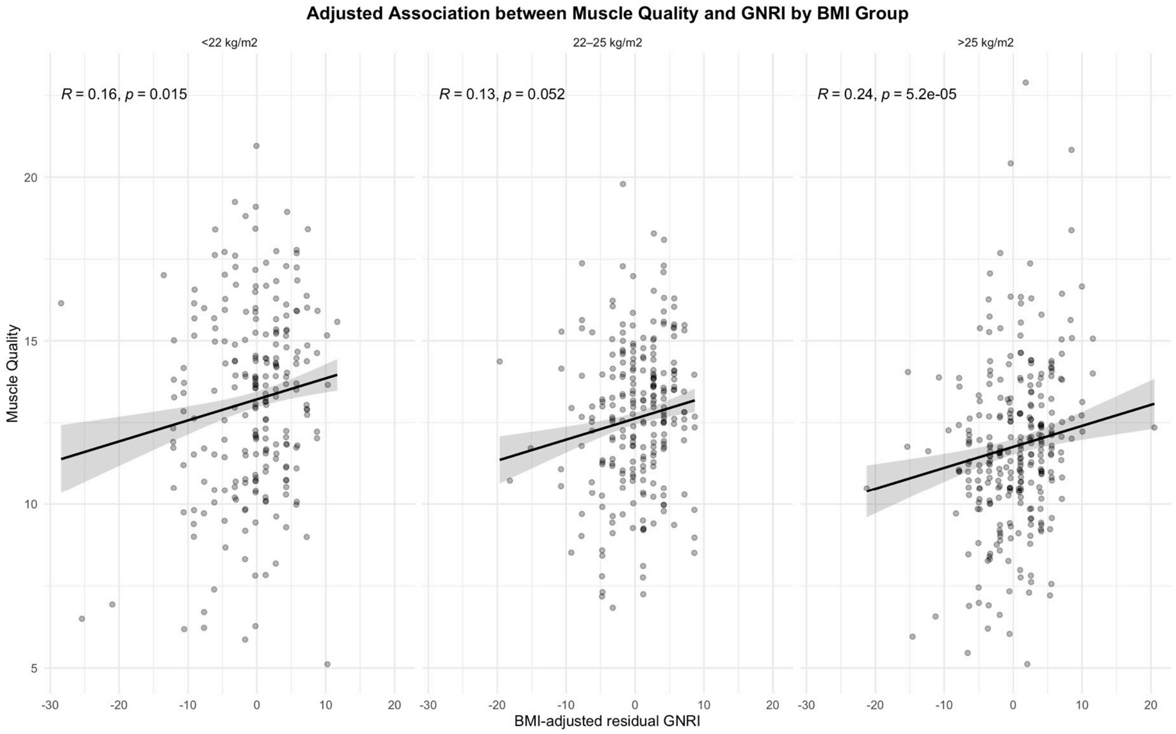

Supplementary Fig. S8 Association between BMI-adjusted residual GNRI and muscle quality stratified by BMI categories. Scatter plots of the relationship between BMI-adjusted residual GNRI and muscle quality across BMI groups: <22 kg/m², 22–25 kg/m², and >25 kg/m².
